# Technique for Determining Bridge Displacement Response Using MEMS Accelerometers

**DOI:** 10.3390/s16020257

**Published:** 2016-02-19

**Authors:** Hidehiko Sekiya, Kentaro Kimura, Chitoshi Miki

**Affiliations:** 1 Advanced Research Laboratories, Tokyo City University, 8-15-1 Todoroki, Setagaya 158-0082, Japan; 2 Urban and Civil Engineering, Tokyo City University, 1-28-1 Tamazutsumi, Setagaya 158-8557, Japan; tyler.durden.kk.999@gmail.com; 3 Tokyo City University, 1-28-1 Tamazutsumi, Setagaya 158-8557, Japan; cmiki@tcu.ac.jp

**Keywords:** bridge health monitoring, microelectromechanical systems accelerometer, free vibration separation method, vehicle detection, measurement error

## Abstract

In bridge maintenance, particularly with regard to fatigue damage in steel bridges, it is important to determine the displacement response of the entire bridge under a live load as well as that of each member. Knowing the displacement response enables the identification of dynamic deformations that can cause stresses and ultimately lead to damage and thus also allows the undertaking of appropriate countermeasures. In theory, the displacement response can be calculated from the double integration of the measured acceleration. However, data measured by an accelerometer include measurement errors caused by the limitations of the analog-to-digital conversion process and sensor noise. These errors distort the double integration results. Furthermore, as bridges in service are constantly vibrating because of passing vehicles, estimating the boundary conditions for the numerical integration is difficult. To address these problems, this paper proposes a method for determining the displacement of a bridge in service from its acceleration based on its free vibration. To verify the effectiveness of the proposed method, field measurements were conducted using nine different accelerometers. Based on the results of these measurements, the proposed method was found to be highly accurate in comparison with the reference displacement obtained using a contact displacement gauge.

## 1. Introduction

Fatigue damage in steel bridges is difficult to detect. Nevertheless, because there is a possibility that fatigue damage will lead to brittle fracture, appropriate inspection and countermeasures are necessary. To detect fatigue damage and take action before brittle fracture occurs, it is important to determine the displacement response of the entire bridge to external forces, in addition to that of each member, and to specify the displacement responses of members that can lead to stress concentrations [1,2]. In particular, the displacement response under live loads is a factor that dominates fatigue damage, and it is important that this can be accurately assessed to ensure steel bridges can be effectively maintained.

One method of directly measuring the displacement is to use fixed reference-based technologies, such as linear variable differential transformers (LVDTs), laser Doppler vibrometers (LDVs) [3], and vision-based systems [4,5,6]. Although these measurements are accurate, they are often impractical because they are costly and difficult to install and, more importantly, they require a fixed reference point, which is often unattainable in full-scale civil structures [7].

As an alternative indirect estimation approach, the use of a microelectromechanical systems (MEMS) accelerometer has recently been proposed for the measurement of the response to an external force [8,9]. Such accelerometers are easy to install on painted metal surfaces using magnets. Furthermore, because MEMS accelerometers are inexpensive and compact, their use in monitoring the health of structures is widespread [10,11,12,13,14,15,16,17,18]. Wireless MEMS accelerometers are easily attachable and detachable. Furthermore, unlike fixed reference-based approaches, the use of MEMS accelerometers does not require a fixed reference point, and thus improved workability is expected.

In theory, the displacement can be calculated from the double integration of the measured acceleration. However, data measured by accelerometers include measurement errors caused by the limitations of the analog-to-digital conversion process and sensor noise. These errors distort the results of the double integration. In addition to the effects of the measurement errors, boundary condition errors also have an adverse effect on the accuracy of the double integration results [19].

Park *et al.*, [9] proposed an initial velocity estimation method in which the average velocity is assumed to be zero. Their method was found to provide highly accurate results when the initial displacement is zero and the times at which vehicles enter and exit the bridge can be determined. However, because a bridge in service is constantly vibrating due to the passing of vehicles, the initial displacement is not always zero. Furthermore, they proposed no method of determining when vehicles enter and exit the bridge. To measure the displacement of a bridge in service, a number of problems remain to be solved.

In theory, the displacement of a bridge can be obtained using an accelerometer or a strain gauge. However, in practice, it is difficult for an accelerometer to accurately measure low-frequency components. Additionally, accurately estimating the displacement using a strain gauge is difficult because the strain is easily affected by measurement noise, particularly in the high frequency range. To compensate for these drawbacks, the indirect displacement estimation using acceleration and strain (IDEAS) method was proposed in previous studies [20,21]. However, it is less time-consuming to attach an accelerometer than a strain gauge.

Nine different accelerometers were selected for use in the present study to compare and validate the accuracy of the displacement results obtained by different accelerometers with different specifications. First, the static characteristics of these nine accelerometers were analyzed. Next, to clarify the vibration characteristics of the test bridge, frequency analysis of the displacement of the test bridge was conducted. Then, based on the free vibration of a bridge in service, a method of calculating the displacement from the measured acceleration was proposed. Finally, to verify the effectiveness of the proposed method, measurements were performed on an actual bridge using the abovementioned nine different accelerometers. Based on the results of these measurements, the proposed method was found to be highly accurate in comparison with the reference displacement using contact displacement gauge.

## 2. Test Bridge and Installation of Accelerometers and Contact Displacement Gauge

**Figure 1:**
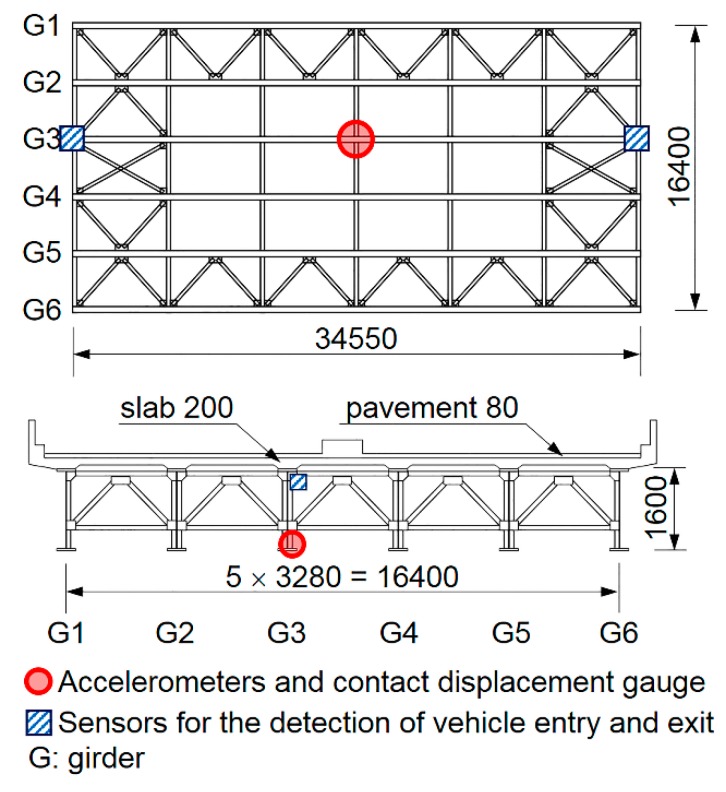
Test bridge used in field measurements (Units: mm).

Measurements of an in-service bridge were conducted using nine different accelerometers and a contact displacement gauge installed at the longitudinal center to determine the displacement response under a live load. The test bridge is shown in Figure 1. It is a 34.55 m-long single-span bridge that consists of six main girders with reinforced concrete (RC) deck plates. It is located in Tokyo and managed by the Metropolitan Expressway Co., Ltd. (Tokyo, Japan).

**Figure 2:**
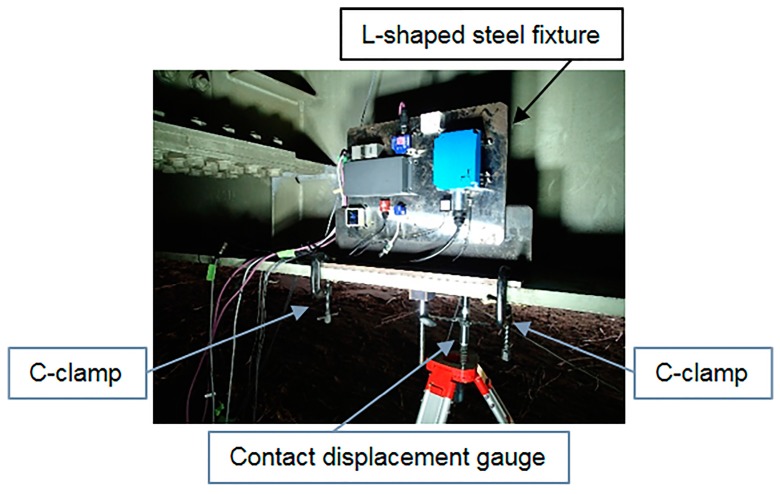
Installation of accelerometers and contact displacement gauge.

The experimental setup of the accelerometers and the contact displacement gauge (CDP-25, Tokyo Sokki) is shown in Figure 2. Each accelerometer was attached by screws to an L-shaped steel fixture that was fixed to the longitudinal center of the lower flange of the main girder (G3) with a C-clamp.

The accelerometers were aligned in the same direction because the performance of some accelerometers in the vertical direction differs from that in the horizontal direction. A contact displacement gauge was also fixed at the longitudinal center of the lower flange of the main girder (G3) to verify the accuracy of the displacement values obtained from the double integration of the measured accelerations. In this test bridge, the contact displacement gauge could be used on the ground because the bridge is located in an earthwork structure that connects to an elevated bridge. The specifications of the contact displacement gauge are listed in Table 1.

### Specifications for Accelerometers Used to Determine Bridge Displacement

The response of a bridge to a live load occurs at low frequencies [22]. In particular, the frequency range for forced displacement due to a live load is frequencies below 1.0 Hz. Assuming the bridge displacement is a superposition of sinusoidal waveforms, the acceleration can be calculated as

(1) $$A=2D{\left(\pi f\right)}^{2}$$

where *A* is the acceleration (m/s^2^), *D* is the total amplitude of the bridge displacement (m), and *f* is the frequency (Hz). Therefore, because acceleration at low frequencies is small, an accelerometer with an inherently high resolution and little sensor self-noise at frequencies below 1.0 Hz must be selected to accurately measure the acceleration; for example, an amplitude of 1.0 mm and a frequency of 0.3 Hz correspond to an acceleration of 0.18 cm/s^2^.

The specifications of the nine different commercial accelerometers, consisting of eight different MEMS accelerometers and a servo-type accelerometer, that were used to determine bridge displacement in the present study are listed in Table 2. However, the noise density, which is an important specification, is only listed for some of the accelerometers in Table 2. This is because in many cases, the noise density is not provided by the manufacturer. In addition, even in cases where the noise density is provided, different manufacturers may use different methods to calculate it. To allow the accelerometers to be compared directly, it is therefore necessary to evaluate the self-noise level for each sensor under the same measurement conditions.

**Figure 3:**
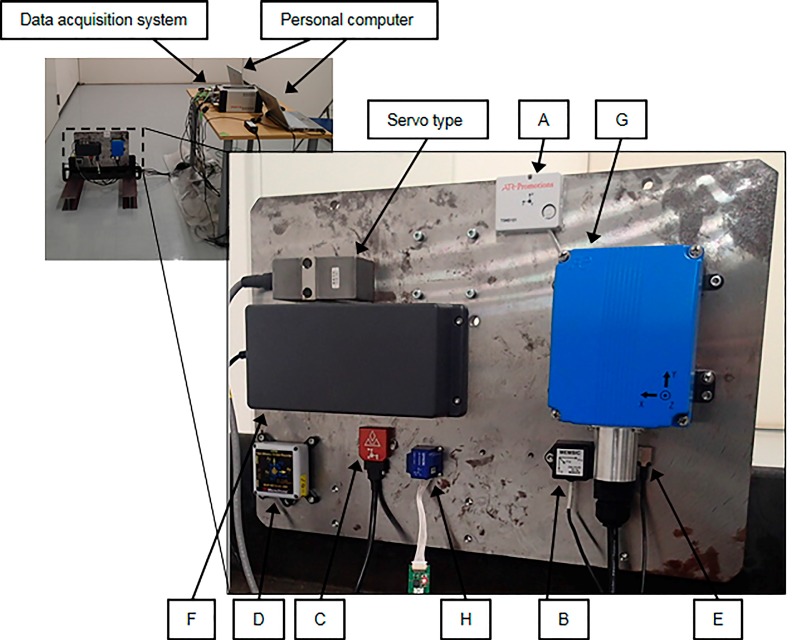
Test setup for analyzing static characteristics of accelerometers listed in Table 2.

To perform such an evaluation, the static characteristics of the accelerometers were measured using the test setup shown in Figure 3. All accelerometers were attached by screws to the same L-shaped steel fixture to ensure the measurement conditions were the same. All measurements were carried out for a duration of 100 s. Although it is generally necessary to eliminate the effects of external factors such as microtremors and thermal sources when evaluating the sensor self-noise, this is very difficult to achieve in practice. Therefore, the noise level for each sensor was taken to be the self-noise for the entire sensor system, including external factors and the effects of analog-to-digital conversion.

**Figure 4:**
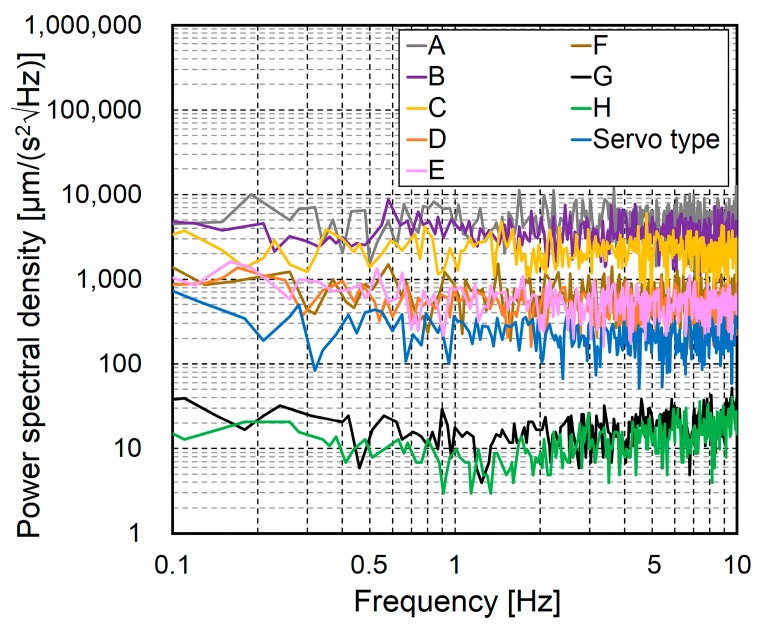
Static characteristics of accelerometers.

Figure 4 shows the static characteristics of the different accelerometers obtained using this method. To present the data independent of the frequency resolution, the power spectral density (PSD) (μm/(s^2^√Hz)) is plotted on the vertical axis. It can be seen that the PSD values at 1.0 Hz are approximately the same as the noise density specifications in Table 2, except for those of Accelerometers G and H.

The higher values obtained for these two accelerometers are thought to be due to microtremors from the floor of the building. At frequencies below 1.0 Hz, all of the accelerometers exhibited very different PSDs, and the PSDs of Accelerometers G and H were found to be at least approximately 20 times smaller than those of the other MEMS accelerometers.

## 3. Free Vibration Separation Method for Determination of Bridge Displacement

In theory, the displacement of a bridge can be calculated from the double integration of the acceleration as:

(2) $$U(T)=U_{0}+V_{0}T_{T}+\int_{T_{0}}^{T_{T}}\int_{T_{0}}^{T_{T}}A(t)dtdt$$

where *U* is the displacement (mm), *A* is the acceleration (mm/s^2^), *V*_0_ is the initial velocity (mm/s), *U*_0_ is the initial displacement (mm), *t* is the time (s), *T*_0_ is the initial time (s), and *T_T_* is the terminal time (s). However, data measured by accelerometers include measurement errors caused by the limitations of the analog-to-digital conversion process and sensor noise. Although these errors distort the results of the double integration, particularly at low frequencies, it is difficult to remove these errors at low frequencies because live loads on the bridge are random and the frequency range for forced displacement due to live loads is also low (below 1.0 Hz). In addition to the effects of the measurement errors, because a bridge in service is always vibrating as a result of the live load conditions, it is difficult to obtain the initial and terminal conditions for the numerical double integration. Therefore, accurately calculating the displacement is difficult.

### 3.1. Frequency Analysis for Determining Dominant Frequency of Displacement under Live Loads

To determine the dominant frequency of the bridge displacement under live loads, frequency analysis was conducted using the displacement record measured in the field with a contact displacement gauge.

**Figure 5:**
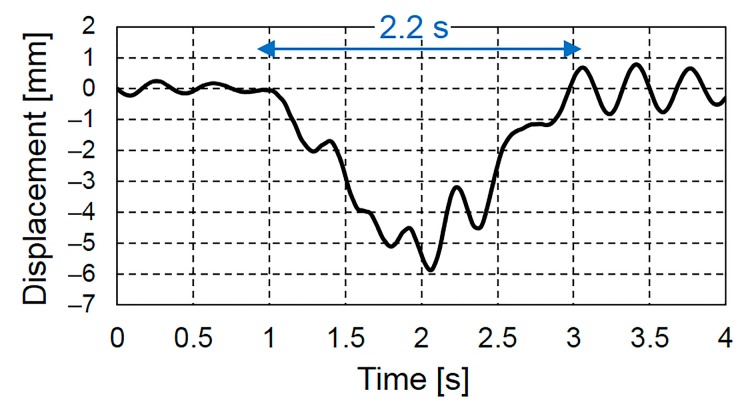
Displacement record at the longitudinal center of the lower flange of the main girder.

Figure 5 shows the displacement measured using the contact displacement gauge for a duration of 4 s at the longitudinal center of the lower flange of the main girder (G3) of the test bridge. Figure 5 shows the typical deflection due to the passage of a vehicle, and the vehicle passing time which depends on the span of the bridge and the vehicle speed, was approximately 2.2 s. Therefore, the frequency of the deflection due to the vehicle weight was approximately 0.5 Hz. In this case, as the length of bridge is 34.55 m, the speed of the vehicle was estimated to be 56.5 km/h.

**Figure 6:**
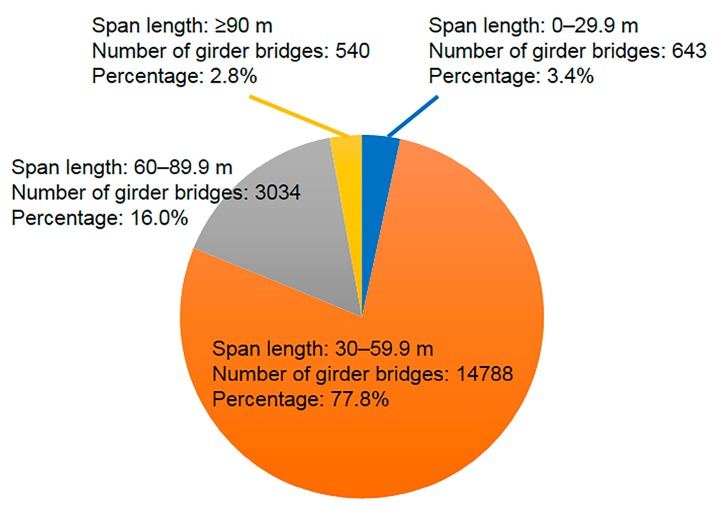
Numbers of girder bridges within different ranges of span lengths.

Figure 6 shows the numbers of girder bridges within different ranges of span lengths. Most girder bridges have span lengths of more than 30 m. In addition, because the maximum allowed speed on expressways in Japan is 100 km/h, the response time should be above 1.2 s, and the frequency should be below 0.9 Hz.

**Figure 7:**
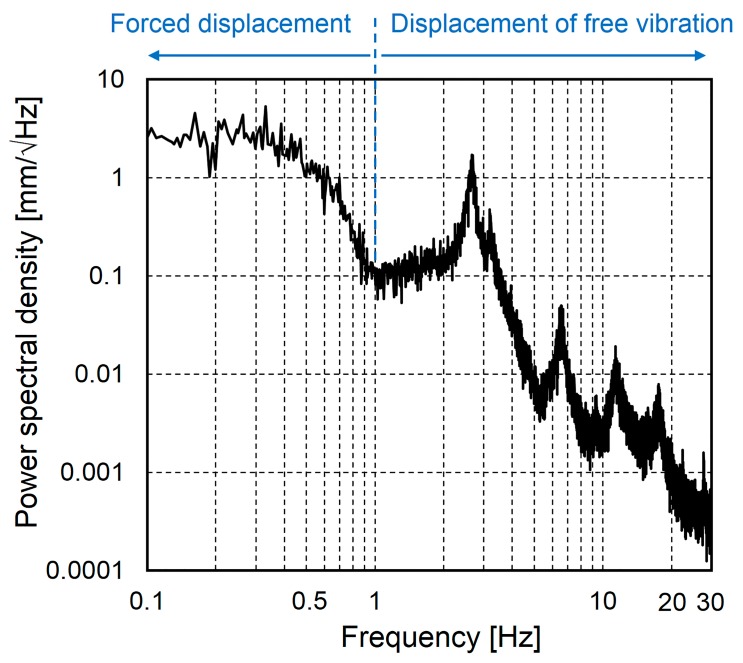
Displacement response spectrum at the longitudinal center of the lower flange of the main girder.

Figure 7 shows the frequency spectrum results of the bridge displacement under live loads for a duration of 2000 s, including the measured displacement shown in Figure 5. Figure 7 shows the dominant frequency range below 1.0 Hz due to the vehicle weight and that above 1.0 Hz due to the free vibration. In this study, only the displacement response at frequencies between 0 and 20 Hz was considered because the power spectral density of the displacement response at frequencies above 20 Hz is negligibly small.

**Figure 8:**
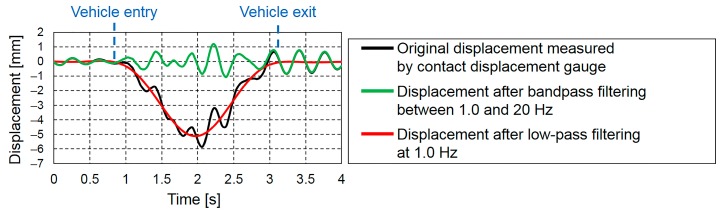
Filtered displacements obtained by separately applying a low-pass filter of 1.0 Hz and a bandpass filter between 1.0 and 20 Hz.

Figure 8 shows the results of filtering the measured displacement shown in Figure 5 by separately applying a low-pass filter of 1.0 Hz and a bandpass filter between 1.0 and 20 Hz. Figure 8 also shows the original measured displacement. The displacement bandpass filtered between 1.0 and 20 Hz, which is the displacement of free vibration, vibrated about the zero-axis before vehicle entry and after vehicle exit. Conversely, the displacement low-pass filtered at 1.0 Hz, which is the forced displacement, started at a displacement of 0 mm when the vehicle entered bridge and returned to a displacement of 0 mm after the vehicle exited the bridge.

### 3.2. Free Vibration Separation Method

Based on the above considerations, we propose a new calculation method, hereafter referred to as the free vibration separation method, the basic concept of which is as follows. First, the initial and terminal conditions for the displacement are determined. Assuming that before vehicle entry and after vehicle exit the bridge is vibrating with sinusoidal oscillations about the zero-axis at its free vibration frequency, which includes the fundamental vibration frequency and higher-order-mode vibration frequencies, the displacement of free vibration can be calculated. Second, the acceleration of the forced displacement component of the bridge displacement while a vehicle is passing over it is numerically integrated to obtain the forced displacement. Finally, the displacement, which includes both the free vibration and forced displacement components, is determined by summing the free vibration displacement and the forced displacement estimated above.

**Figure 9:**
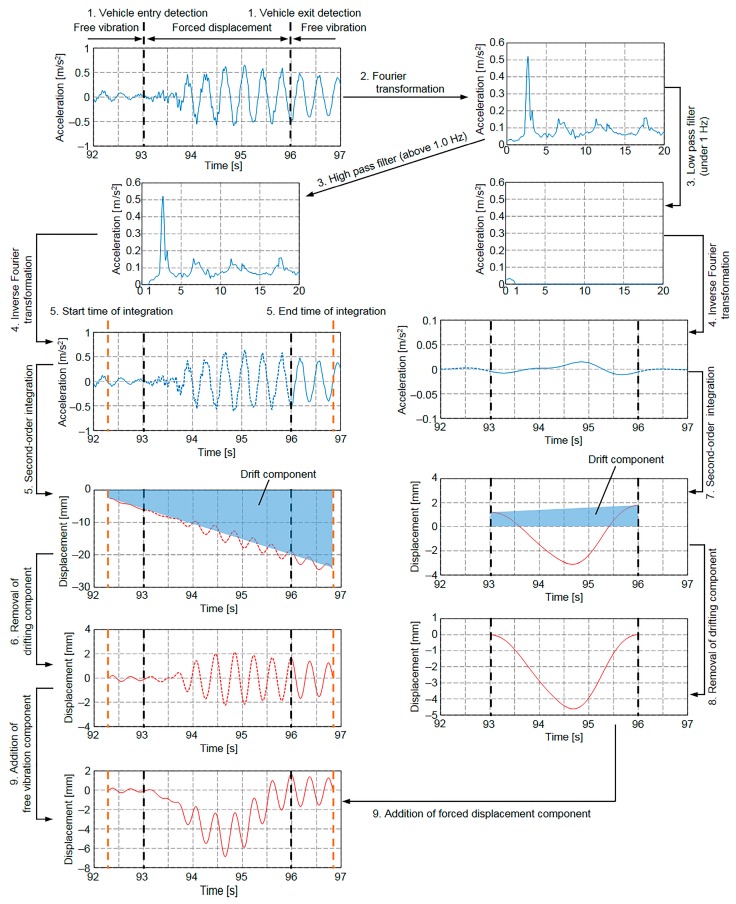
Application of proposed free vibration separation method of determining bridge displacement.

The procedure of the free vibration separation method is shown in Figure 9. It consists of the following steps.

1. The free and forced vibration regions are separated by detecting vehicle entry and exit.
2. The acceleration is transformed from the time domain to the frequency domain by Fourier transform.
3. By removing the frequencies below 1.0 Hz to eliminate the effect of forced displacement during vehicular passage, the acceleration of free vibration in the frequency domain is estimated. In the same manner, by removing the frequencies above 1.0 Hz to eliminate the effect of free vibration, the acceleration of forced displacement in the frequency domain is estimated.
4. The accelerations of free vibration and forced displacement in the time domain are estimated by taking the inverse Fourier transform of the accelerations obtained in Step 3.
5. Assuming the bridge is vibrating with sinusoidal oscillations about the zero-axis, the acceleration of free vibration should be zero when the displacement of free vibration is zero. Therefore, the acceleration of free vibration obtained in Step 4, which excludes the effects of vehicular forced displacement, is numerically integrated twice from the time when the acceleration of free vibration is zero before vehicle entered to the time when the acceleration of free vibration returns to zero after vehicle exited to obtain the displacement.
6. Because of the measurement error in the measured acceleration, which is caused by sensor noise and the limitations of analog-to-digital conversion, integration error, and the constants of integration, the initial and terminal displacements of the integrated displacement are not equal to zero. To make the initial and terminal displacements equal to zero, the drift component is subtracted from the integrated displacement.
7. The acceleration of forced displacement obtained in Step 4, which excludes the effects of free vibration, is numerically integrated twice to obtain the displacement. The lower and upper limits of the integration are the times when the first vehicle enters the bridge and the last vehicle exits the bridge, respectively.
8. Because of the measurement error in the measured acceleration, which is caused by sensor noise and the limitations of analog-to-digital conversion, integration error, and the constants of integration, the initial and terminal displacements of the integrated displacement are not equal to zero. In the same manner as in Step 6, the drift component is subtracted from the integrated displacement to make the initial and terminal displacements equal to zero.
9. By linearly summing the displacements of free and forced vibration, the total displacement, which includes free and forced vibration components, is estimated.

## 4. Field Measurements

As described above, measurement errors distort the results of double integration. To minimize such distortion, an accelerometer with an inherently high resolution and little sensor self-noise should be selected, and the free vibration separation method described above should be applied to the numerical integration process. To verify the effectiveness of this approach, measurements were conducted on an actual bridge using nine different accelerometers.

### 4.1. Detection of Vehicle Entry and Exit

**Figure 10:**
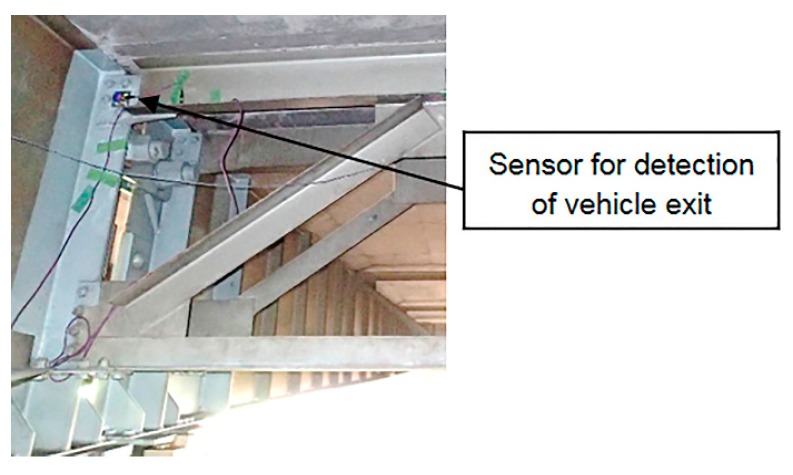
Installation of accelerometer for detection of vehicle exit.

**Figure 11:**
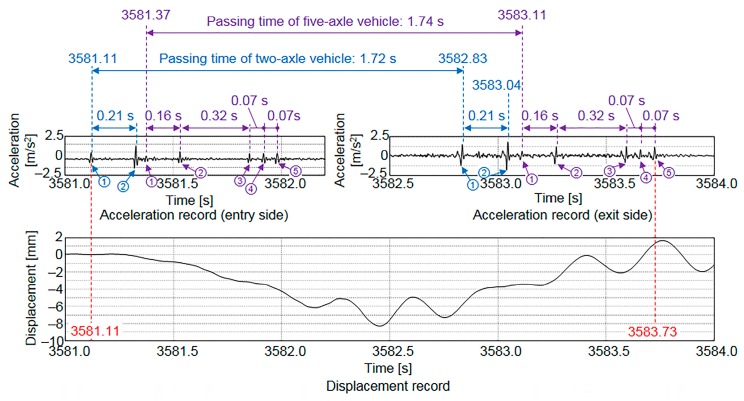
Times of vehicle entry and exit based on acceleration record.

To detect vehicle entry and exit, instead of applying the conventional method using a strain gauge [23], the acceleration was measured using accelerometers at the vertical stiffeners on both longitudinal edges of the main girder. The specifications of the accelerometers are listed in Table 3. The sampling frequency for vehicle detection was 500 Hz. The experimental setup for the accelerometer used to detect vehicle exit is shown in Figure 10. Figure 11 shows the acceleration record and the displacements measured using the contact displacement gauge. The acceleration record revealed the passage of one vehicle with two axles and another vehicle with five axles. Because the passing times of the two vehicles (1.72 and 1.74 s) were different, the results indicated that two vehicles were passing over the bridge. Furthermore, because the distances between the peaks in the acceleration record at the entry and exit sides were equal, it was concluded that the two vehicles passed over at constant speeds. Because the length of the test bridge is 34.55 m, the vehicle speeds, which were 72.3 and 71.5 km/h for the two- and five-axle vehicles, respectively, could be estimated. The entry time and the exit time for the two-axle vehicle (3581.11 s, 3583.04 s) and the entry time and the exit time for the five-axle vehicle (3581.37 s, 3583.73 s) are indicated in Figure 11.

### 4.2. Displacement Results Obtained Using Proposed Free Vibration Separation Method at Longitudinal Center

**Figure 12:**
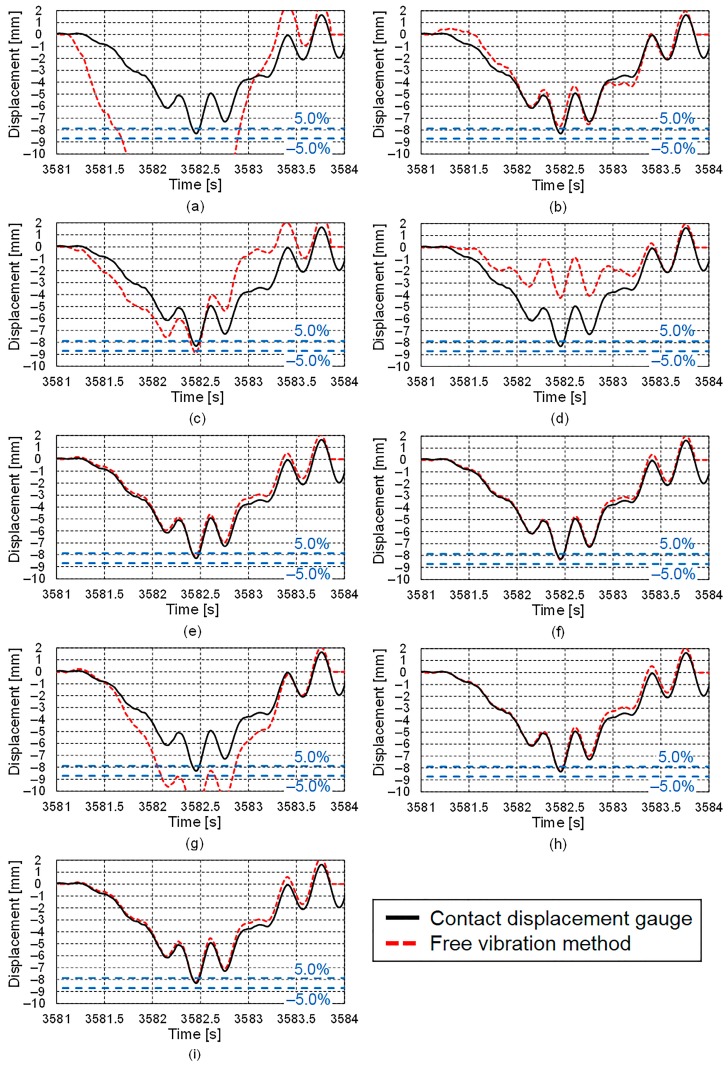
Displacement responses obtained using proposed free vibration separation method: (**a**) Accelerometer A; (**b**) Accelerometer B; (**c**) Accelerometer C; (**d**) Accelerometer D; (**e**) Accelerometer E; (**f**) Accelerometer F; (**g**) Accelerometer G; (**h**) Accelerometer H; (**i**) Servo-type accelerometer.

The displacement results obtained using the proposed free vibration separation method are shown in Figure 12.

The starting (3581.11 s) and ending (3583.73 s) times, which were obtained from the acceleration record at the vertical stiffener, were used as the limits of the numerical integration. By reducing the interval between the starting and ending times used in the integration of the measured acceleration, the integration error due to the measurement error of the measured acceleration can be diminished.

The results for Accelerometers E, F, and H and the servo-type accelerometer, all of which had resolutions of less than 9.8 μm/s^2^ and sensor noise levels of less than 2.0 mm/(s^2^√Hz), and the results obtained using the contact displacement gauge differed by 5.0% or less at the maximum displacement point. However, the results for Accelerometer G, the sensor noise level of which was approximately the same as that of Accelerometer H, were totally different from those obtained using the contact displacement gauge. The reason for this is thought to be that the measurement error due to the aliasing of the measured acceleration distorts the results. Furthermore, the results for Accelerometer D, the sensor noise level of which is approximately the same as those of Accelerometers E and F, were also markedly different from those obtained using the contact displacement gauge. The reason for this is thought to be that the resolution of Accelerometer D is too large to accurately measure the response at frequencies lower than 1.0 Hz. For example, the acceleration is 490 μm/s^2^ for an amplitude of 0.1 mm and a frequency of 0.5 Hz. The results for Accelerometers A, B, and C and those obtained using the contact displacement gauge differed by more than 5.0% at the maximum displacement point. A low-frequency component was present in the results for these accelerometers because of the measurement error due to the limitations of the analog-to-digital conversion process and their sensor noise levels, which were more than 50 times that of Accelerometer H.

### 4.3. Validation of the Consistency of the Results Using Free Vibration Separation Method to Determine Displacement Response

**Figure 13:**
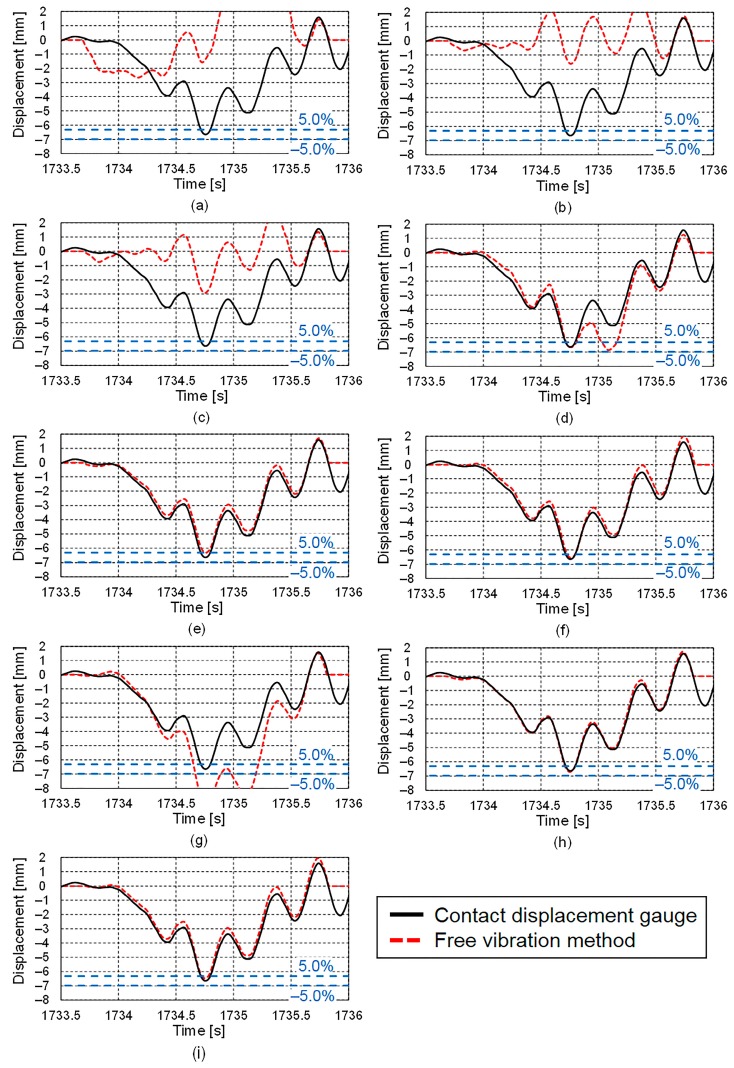
Displacement responses obtained using proposed free vibration separation method: (**a**) Accelerometer A; (**b**) Accelerometer B; (**c**) Accelerometer C; (**d**) Accelerometer D; (**e**) Accelerometer E; (**f**) Accelerometer F; (**g**) Accelerometer G; (**h**) Accelerometer H; (**i**) Servo-type accelerometer.

In order to validate the consistency of the results using the free vibration separation method to determine displacement response, a displacement response for a time different from the time in Figure 12 is determined by the free vibration separation method using nine different accelerometers. The starting (1733.65 s) and ending (1735.69 s) times, which were obtained from the acceleration record at the vertical stiffener in the same manner as shown in Figure 11, were used as the limits of the numerical integration. The displacement results obtained using the proposed free vibration separation method are shown in Figure 13. As with the result in Figure 12, the results for Accelerometers E, F, and H and the servo-type accelerometer, all of which had resolutions of less than 9.8 μm/s^2^ and sensor noise levels of less than 2.0 mm/(s^2^√Hz), are very similar to the results obtained using the contact displacement gauge. On the other hand, the results of Accelerometers A, B, C, and G were markedly different from those obtained using the contact displacement gauge. Although the result of Accelerometer D give accurate peak displacement, the result of the displacement did not coincide, partially because of a low-frequency component due to the measurement error due to limitations of the analog-to-digital conversion process and its sensor noise levels.

## 5. Conclusions

In the present study, the static characteristics of nine different commercial accelerometers were first analyzed. Next, to clarify the vibration characteristics of the test bridge, frequency analysis of the displacement of the test bridge was conducted. Then, based on the free vibration of an in-service bridge, a method of calculating the displacement from the measured acceleration was proposed; this method is called the free vibration separation method. To determine the initial and terminal times for use as the lower and upper limits of the numerical integral, vehicle entry and exit times were detected using the response of the measured acceleration at the vertical stiffeners on both longitudinal edges of the main girder. Finally, measurements were performed on an actual bridge using nine different accelerometers to verify the effectiveness of the proposed method. The conclusions of the present study are as follows.

- Based on the free vibration of a bridge in service, the free vibration separation method for determining the bridge displacement was proposed.
- To apply the proposed method, the vehicle entry and exit times must be detected. In this study, this was achieved using the acceleration measured at a vertical stiffener of the main girder. Furthermore, using the distance between peaks in the acceleration record, the fact that multiple vehicles were passing over the bridge was able to be determined. In addition, using the distance between peaks in the acceleration record, the speeds of the vehicles were able to be estimated.
- To verify the effectiveness of the proposed method in determining the bridge displacement under a live load, field measurements were carried out using nine different accelerometers. Implementing the proposed method using an accelerometer with a resolution of less than 9.8 μm/s^2^ and a sensor noise level of less than 2.0 mm/(s^2^√Hz), the calculated displacement response was found to be in good agreement with the reference displacement measured by the contact displacement gauge.
- In order to validate the consistency of the results using free vibration separation method to determine displacement response, the displacement at different time is determined. As a result, by using accelerometer with a resolution of less than 9.8 μm/s^2^ and a sensor noise level of less than 2.0 mm/(s^2^√Hz), a result which is similar to the reference displacement measured by the contact displacement gauge could be determined at a different time. The consistency of the results could thus be demonstrated.

In the case of a short vehicle passing time, particularly for the passage of a single vehicle, the proposed method with high resolution and low sensor noise shows good agreement with the reference displacement measured by the contact displacement gauge. However, some traffic conditions may cause serious problems. For example, the adverse effect of the measurement error on the integration results become large when multiple vehicles continuously pass over the bridge because the integration time of the forced displacement is large. Such cases require careful attention.

## Figures and Tables

**Table 1:** Specifications of contact displacement gauge to verify displacement obtained from acceleration.

Contact Displacement Gauge	Capacity (mm)	Sensitivity (×10^−6^ Strain/mm)	Nonlinearity (mm)	Sampling Frequency (Hz)
CDP-25 (Tokyo Sokki)	0–25	500	0.1% RO	100

**Table 2:** Specifications of commercial accelerometers used to determine bridge displacement.

Accelerometer	Acceleration Range (m/s^2^)	Frequency Bandwidth (Hz)	Sampling Frequency (Hz)	Resolution (μm/s^2^)	Noise Density (μm/(s^2^√Hz))
A	±19.6	0.1–500	100	588	3923
B	±16.7	0.1–50	100	4070	-
C	±156.9	0.1–260	100	4786	3923
D	±19.6	0.1–100	200	745	-
E	±19.6	0.1–20	100	5.9	-
F	±49.0	0.1–330	153.75	3.7 × 10^−2^	539
G	±19.6	0.1–50	200	196	6.4
H	±49.0	0.1–20	100	9.8	7.8
Servo type ASQ-D (Kyowa)	±19.6	0.1–20	100	2.0	-

**Table 3:** Specifications of accelerometers used to detect vehicle entry and exit.

Accelerometer	Acceleration Range (m/s^2^)	Frequency Bandwidth (Hz)	Sampling Frequency (Hz)	Resolution (μm/s^2^)	Noise Density (μm/(s^2^√Hz))
M-G550-PC (SEIKO EPSON)	±29.4	0.1–148	500	1226	981

## Abbreviations

The following abbreviations are used in this manuscript:

MEMS: microelectromechanical systems
LDVT: linear variable differential transformer
LDV: laser Doppler vibrometer
IDEAS: indirect displacement estimation using acceleration and strain
RC: reinforced concrete
PSD: power spectral density
